# A Spiritually-Based Text Messaging Program to Increase Cervical Cancer Awareness Among African American Women: Design and Development of the CervixCheck Pilot Study

**DOI:** 10.2196/formative.8112

**Published:** 2018-03-29

**Authors:** Daisy Le, Linda Aldoory, Mary A Garza, Craig S Fryer, Robin Sawyer, Cheryl L Holt

**Affiliations:** ^1^ Department of Behavioral and Community Health School of Public Health University of Maryland College Park, MD United States; ^2^ Department of Communication University of Maryland College Park, MD United States

**Keywords:** short message service, text messaging, African Americans, women’s health, cervical cancer, health status disparities, pap test, cancer screening, health information technology, spirituality, community-based participatory research

## Abstract

**Background:**

Although Hispanic women have the highest cervical cancer incidence rate, African American women account for a disproportionate burden of cervical cancer incidence and mortality when compared with non-Hispanic white women. Given that religion occupies an essential place in African American lives, delivering health messages through a popular communication delivery channel and framing them with important spiritual themes may allow for a more accessible and culturally appropriate approach to promoting cervical cancer educational content to African American women.

**Objective:**

The aim of this paper was to describe the design and development of the CervixCheck project, a spiritually based short message service (SMS) text messaging pilot intervention to increase cervical cancer awareness and Papanicolaou test screening intention among church-attending African American women aged 21 to 65 years.

**Methods:**

Through focus group interviews (n=15), formative research was conducted to explore facilitators, motivators, and barriers to cervical cancer screening. The interviews were also used to identify logistical factors that should be considered when developing the CervixCheck intervention. Culturally appropriate and spiritually grounded SMS text messages were developed based on the analysis of focus group data and the review of previous studies that incorporated technology into health behavior change interventions. After the CervixCheck intervention was developed, cognitive response interviews (n=8) were used to review the content of the SMS text messaging library, to ensure that the content was acceptable and understandable, particularly for church-attending African American women aged 21 to 65 years.

**Results:**

Design and development of the SMS text messages involved consideration of the content of the messages and technological specifications. Focus group participants overwhelmingly reported cell phone use and an interest in receiving spiritually based SMS text messages on cervical cancer prevention and early detection. Findings from the cognitive response interviews revealed that the content of the SMS text messaging library was acceptable and understandable with the target population. The revised SMS text messaging library currently includes 22 messages for delivery over 16 days, averaging 11 texts per week, with no more than two messages delivered per day. Initial usability testing also showed early feasibility.

**Conclusions:**

The design and development of the CervixCheck intervention provides important insight into what may be considered an overlooked minority population and missed opportunity in health information technology research. With increased internet penetration through the use of mobile phones, it is appropriate to investigate the viability of technology as a means to reach minority communities and to reduce health disparities.

## Introduction

### Background Information

Although cervical cancer incidence and mortality rates have drastically decreased in the United States over the last few decades [1,2], particularly because of Papanicolaou (Pap) testing, some populations still continue to bear a larger burden of the disease [1,3,4]. On a national level, African American women experience the second highest incidence rate of cervical cancer (11.4/100,000) and the highest death rate (4.9/100,000) [2]. When compared with white women in the general population, African American women have a 34% higher incidence of cervical cancer and are twice as likely to die of the disease in the United States [1,3,4].

Despite the similarities in cancer screening habits between African American and white women, the former is still more likely to be diagnosed with advanced stage of cervical cancer [4-6]. Although these racial differences are evident, several studies have indicated that race is not a predictor of cervical cancer; the effect of race diminishes as other factors such as education and socioeconomic status are taken into account [5,7]. A Massachusetts study found that these racial differences could possibly be caused by inadequate education for patients and providers, fear and mistrust of the health care system, cultural differences in health-seeking behaviors, and challenge in diagnostic testing after an abnormal Pap test [8,9]. Another study by Schwartz and colleagues (2003) found that socioeconomic status accounts for most of the diagnosis stage disparity between African Americans and whites for cervical cancer [10]. Other studies have attributed the differences in cervical cancer mortality rates between African American and white women to the quality of screening and follow-up after abnormal screening that African American women receive [4] and to higher disease stage upon diagnosis [4-6,11].

It has been evident that in the last three decades that there has not only been an increase in mass education both in rural [12] and urban areas [13] but reductions in financial difficulties with Pap tests covered by the majority of health insurance plans including Medicaid as well. Regardless of these reductions in educational and financial barriers, disparities in cervical cancer screening have persisted, with African American women being one of the populations that continue to bear a disproportionate burden of the disease. Fortunately, cervical cancer is one of the most preventable types of cancer, and women in the age range of 21 to 65 years can get screened for it with a routine Pap test [3,4]. Current screening guidelines recommend that women should have regular cervical cancer screening starting at the age of 21 years till at least the age of 65 years. For women of average risk, current screening guidelines recommend getting screened with the Pap test every 3 years. For women ages 30 years and older, cotesting (a Pap test with human papillomavirus [HPV] test) every 5 years is also recommended (either that or routinely getting the Pap tests every 3 years should still continue) [14,15]. Identifying opportunities to improve Pap test screening utilization and adherence are critical to reduce the cancer burden in African American women [3,4].

### Religiosity or Spirituality and Health in the African American Community

Screening and early detection, particularly by identifying opportunities to improve Pap test screening utilization, are critical components in eliminating the aforementioned disparities in health outcomes for African American women [3,4]. There are a number of social and cultural factors that relate to prevention and screening behaviors that impact cancer mortality rates. Religious involvement is one of these factors [16-21]. Extensive research has shown that religious involvement plays an important role in the African American community [16-22]. In particular, older African American women have been found to be more religiously involved than other groups [17,19].

The relationship between religiosity or spirituality and health has gained much consideration in recent scientific literature, as well as amid lay audiences [23-26]. Research has extensively examined the relationship between religious involvement and a wide variety of physical and mental health outcomes [27]. These relationships are generally agreed to be positive in nature [28,29], suggesting a beneficial impact on health. Theoretical models and literature proposes that the reason religiously involved individuals tend to have good health outcomes is because they have healthy lifestyles and behaviors that align with their religious beliefs. This perceived religious effect on health behavior [30] may reflect religious doctrine, or the common belief that the body is the temple of the holy spirit [31], and include greater engagement in health behaviors such as physical activity (PA), diet, and screening [29], while avoiding behaviors such as drinking alcohol excessively and risky sexual practices [28,31].

Religious involvement has been associated with cancer beliefs, screening, risk, and prevention behavior and has great potential for use in the development of cancer prevention and screening communication interventions for this group [16-21]. Due to the popularity of church-based cancer screening programs for African Americans and the well-established association between religious involvement and health in the literature [16-21,32,33], it is logical to consider health promotion programs that engage faith-based institutions and that are spiritually based to address the health needs of the African American community. Given the relatively high relevance and frequency that religion plays in the daily lives of African American women [34], it is important to explore how religious beliefs and behaviors may influence an individual’s perception, initiation, engagement, and participation in cervical cancer screening prevention.

### Text Messaging as an Intervention Communication Delivery Channel

Mobile phone technology represents a nearly universal form of communication and is a promising new medium of intervention delivery in health research [15]. The National Institutes of Health (NIH) Consensus group defines mobile health (mHealth) as the use of mobile and wireless devices to improve health outcomes, health care services, and health research [35,36]. Literature relating to short message service (SMS) text messaging has revealed that SMS text messages may be an effective strategy for stimulating behavior change or supporting behavioral interventions [37-39]. Periodic cues through this type of communication medium have been also been found to be effective in reinforcing healthy behaviors [37], including sensitive health-related issues such as sexually transmitted infection (STI) prevention [40-42]. For example, combined social media and SMS text messaging interventions have been used to successfully promote weight loss [43-45] and various health behaviors (ie, PA and dietary behaviors) [45-47] in previous research and have several benefits compared with mailed print-based or traditional face-to-face health interventions. Specifically, SMS text message reminders have established popularity with patients and have been shown to be more cost-effective than paper- or telephone-based reminder strategies [48]. Such evidence implies that SMS text messaging can be an effective medium to deliver health information and promote preventive behaviors.

Results across recent studies on cancer-specific SMS text messaging interventions for minority populations have already demonstrated both participant interest in SMS reminders for cancer screening appointments and a positive effect on screening rates [49,50]. For example, in a study of younger Korean American women where a 7-day SMS text messaging program for cervical cancer screening was created to stimulate increases in knowledge and behavior pre-post intervention, preliminary results showed that 23% of participants received a Pap test after the intervention [51]. In addition, in a pilot single-group study with a pre-post design, Spark and colleagues also yielded positive results from their cancer-specific SMS text messaging intervention [52]. In this study, Spark and colleagues investigated whether a 6-month extended contact intervention delivered through highly tailored SMS text messages would support long-term weight loss, PA, and dietary behavior change in breast cancer survivors (vs usual care). Results from this study supported the feasibility, acceptability, and provided preliminary evidence on efficacy of an SMS text message–delivered extended contact intervention to promote the maintenance of weight loss and PA among a predominately older female subgroup. On the basis of a recent systematic review of SMS text messaging interventions on cancer screening rates [53], the absolute screening rates for SMS text message recipients were found to be 0.6% to 15.0% higher than for controls, whereas the unadjusted relative screening rates for individuals who received SMS text messages were 4% to 63% higher compared with controls. It was also reported that SMS text messaging interventions seemed to moderately increase screening rates for breast and cervical cancer, while having a smaller effect on colorectal cancer screening. Benefits were shown across several countries, including non-English-speaking and resource-poor populations.

Although the use of SMS text messaging interventions in cancer prevention and control is growing in the general population and is emerging among minorities and the medically underserved, the number of studies that specifically focus on African Americans remain limited. In one study of predominately African American women, findings revealed that this group was receptive to receiving SMS text messages that focus on cancer and health information [54]. Similarly, some feasibility and positive acceptability of using SMS text messaging in a prostate cancer research project were also found for older African American men in the age range of 40 to 69 years [39]. In the Men’s Prostate Awareness Church Training project, SMS text messages were added to a men’s health intervention that aimed to increase informed decision making on prostate cancer screening. To the authors’ best knowledge, and aside from Yuan and colleagues’ work where study findings are still pending [55], this study was the only research found to date that utilized SMS text messaging as a means to reach mature African American men. Moreover, there does not appear to be previous research using this technology to increase cervical cancer prevention among African American women, particularly as a stand-alone intervention that uses a spiritually based approach. This study will provide important insights regarding the feasibility, acceptability, and initial efficacy of a spiritually based SMS text messaging educational intervention in the promotion of cervical cancer prevention information for what may be an overlooked minority population and missed opportunity in health information technology research [45,56-60].

### Active Users of Text Messaging

With respect to SMS text messaging behavior, there are several groups that text on a daily basis at higher-than-average levels [61]. On the basis of cell phone owners who use SMS text messaging among a sample of 2277 adults (18 years and older) who were telephone interviewed by Princeton Survey Research Associates International from April 26, 2011 to May 22, 2011, the average number of SMS text messages sent or received on a normal day is approximately 41.5, with the median user sending or receiving 10 SMS text messages per day. Although women send and receive SMS text messages more frequently (mean 42.0/day; median 15/day) than men (mean 40.9/day, median 10/ day), African Americans send and receive SMS text messages on a more frequent basis (mean 70.1/day; median 20/day) than their non-Hispanic white counterparts (mean 31.2/day, median 10/ day) [61]. Although the use of SMS text messaging decreases by age group, it is evident that a majority (73%) of American adults use this mobile-based technology to communicate. The higher-than-average levels of SMS text messaging in women (mean 42.0/day; median 15/day) and African Americans (mean 70.1/day; median 20/day), as well as the indication that most Americans across all age groups engage in some frequency of SMS text messaging (for those aged 18-64 years: mean>11.4/day; median>3/day), suggest the potential suitability of delivering a SMS text messaging–based health intervention to African American women aged 21 to 65 years.

SMS text messaging was selected as the primary delivery channel for the current intervention because of its popularity and high use among African American women. Nationally representative data show that African American adults are more likely to own a mobile phone (87%) when compared with non-Hispanic whites (80%) [62]. Additionally, African Americans, in general, are more likely than their non-Hispanic white counterparts to use their mobile phones to send and receive SMS text messages [56,63-65] and to access social media websites (ie, Twitter, Facebook, etc) [56,65,66]. The most recent data from the Pew Internet and American Life Project reports that 80% of African Americans and 80% of all women use mobile cell phones for sending or receiving SMS text messages [64]. The low cost and widespread use of mobile phones and the convenience of SMS text messaging further suggest the potential suitability of employing this type of mobile-based medium for delivering health promotion interventions to African American women.

### This Study

The purpose of this three-phased multiple methods study was to develop, pilot-test, and evaluate the feasibility, acceptability, and initial efficacy of “CervixCheck,” a spiritually based SMS text messaging intervention for the promotion of cervical cancer early detection among church-attending African American women in the age range of 21 to 65 years. Given the high levels of technology use in African Americans and substantial evidence suggesting that technology-based health promotion efforts are effective for stimulating behavior change and supporting behavioral interventions [45,56,57,60], the minimal previous research on SMS text messaging as a means to promote cervical cancer early detection represents a missed opportunity to reducing cervical cancer mortality rates in this population. Although there is a growing body of literature reporting positive outcomes of SMS text message–based communication with STIs [40-42] and cancer prevention [39,55,67-70], few focus on cervical cancer screening [51] or African American women [54], and none of these SMS text message interventions for cervical cancer prevention focus on African American women or use a spiritually based approach, making this intervention unique. Spiritually based SMS text messages on health allows for a more culturally appropriate technology-based approach to promoting cervical cancer early detection educational content to African American women and can potentially serve as an effective intervention strategy to reach this population.

This paper reports on the formative research (phase 1) that was conducted to inform the development of the CervixCheck intervention. We also report on the iterative process of intervention and delivery system development (phase 2). Phase 3 of this study, reported elsewhere, was used to determine the feasibility, acceptability, and initial efficacy of SMS text messages in the delivery of cervical cancer early detection educational content to African American women.

## Methods

### Ethical Approval

This research was reviewed and approved according to the University of Maryland Institutional Review Board’s procedures for research involving human subjects (866903-1).

### Study Overview and Design

**Figure 1:**
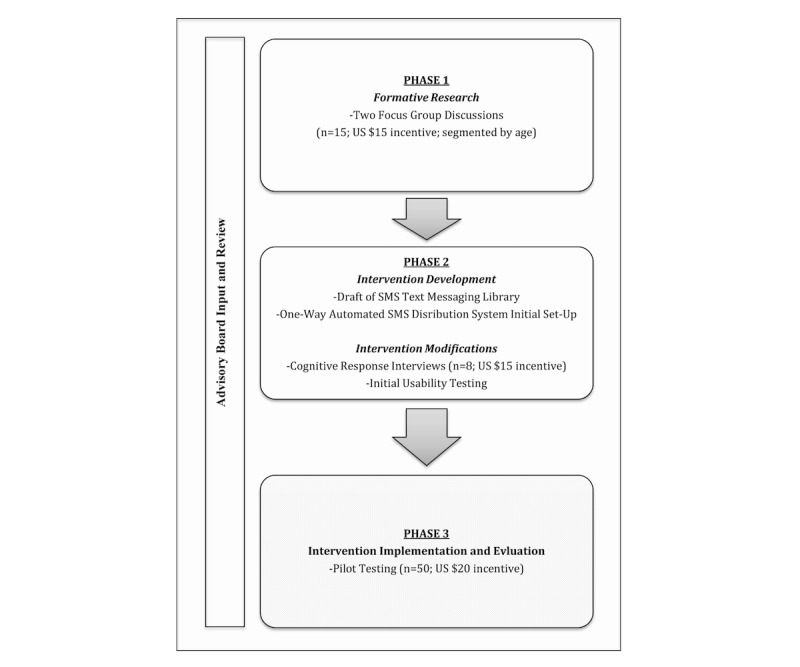
Intervention development process. Phase 3 of this study is reported elsewhere.

The CervixCheck project was conducted in three phases. Phases 1 and 2, completed in February and March 2016 and reported in this paper, involved the development and initial usability testing of a SMS text messaging intervention and automated distribution system (Figure 1).

First, two semistructured focus group discussions (n=15) were conducted to explore knowledge, beliefs, attitudes, barriers, facilitators, and motivators in cervical cancer screening for church-attending African American women in the age range of 21 65 years. They were also used to identify factors (eg, message content and timing) that should be considered when developing a spiritually based SMS text messaging intervention targeted at women such as themselves. Next, culturally appropriate and spiritually grounded SMS text messages were developed based on the analysis of our focus group data, feedback from our community advisory board, and review of previous studies that incorporated technology into health behavior change interventions [37-48,51-55,67-70]. Finally, after the CervixCheck intervention was designed and developed, cognitive response interviews (n=8) were used to assess the content of the SMS text messaging library. The compilation of SMS text messages in the library database were ultimately refined and incorporated into an automated SMS distribution system and was piloted for feasibility, acceptability, and initial efficacy in phase 3 (reported elsewhere).

### Community-Engaged Approach

Community-engaged research requires partnership development, collaboration, and negotiation, as well as the commitment from both the community and academic researchers to addressing local health issues. Community-engagement activities involved in this study included the following: (1) conducting formative research for intervention development; (2) setting the study in the community, at an agreed-upon location and time of convenience to the study participants; (3) securing buy-in and recruitment or retention support from pastors and community health advisors; (4) forming a community advisory board; and (5) building in and carrying out member checks throughout the study.

### Community Advisory Board

Members of the priority population were identified and approached by the principal investigator (PI) to serve on a community advisory board. The six board members were in the age range of 22 to 61 years. All members were church-affiliated African American women. Four indicated that they either were currently serving or had previously served in a leadership capacity within their local congregations (eg, head of a health or women’s ministry and previous community health advisor). This advisory board contributed to the development of the intervention materials and provided recommendation for other aspects of the project (eg, recruitment strategies and SMS text messaging content and delivery time or format).

### Participant Eligibility, Recruitment, and Sample

African American women aged 21 to 65 years were recruited from the research team’s professional networks and from current collaborations with faith-based organizations in Prince George’s County, MD. Participants were also recruited through social media (Facebook), emails sent to numerous listservs, flyers posted at churches, workplaces and campuses, and snowballing techniques during the months of February 2016 and March 2016. Those responding to the recruitment materials were screened for eligibility before being scheduled for either a focus group discussion or a cognitive response interview.

Phases 1 and 2 of the CervixCheck intervention were limited to self-identifying church-attending African American women who were in the age range of 21 and 65 years and who had indicated that they reside within Prince George’s County, MD. Only individuals who had not had a past medical history of cervical cancer or hysterectomy (ie, the surgical removal of the corpus uteri) were considered for the study. Eligible participants who were interested in participating in phase 1 or 2 of the intervention must have also been willing to discuss topics surrounding culture, technology use, and cervical cancer prevention and education. Participants were expected to meet in person at an agreed-upon location and time of convenience. This often meant meeting in the community during the evenings and weekends to enable those who worked during the day to participate. Individuals in the focus group discussions and cognitive response interviews received US $15 each for their involvement. Participants were only eligible to participate in either the focus group discussions or the cognitive response interviews.

### Focus Group Discussions

Before intervention development, formative research was conducted through two semistructured focus group discussions to inform the development of the CervixCheck intervention. The discussions qualitatively explored African American women’s beliefs and attitudes about cervical cancer prevention and education and assisted in determining factors that would be needed to be considered when developing the spiritually based SMS text messaging educational intervention aimed at increasing Pap test screening intention in church-attending African American women. The focus groups were segmented by age (21-35 years and 36-65 years) to facilitate more open discussion and identify any age group differences. The sessions followed a semistructured format and lasted approximately 90 min. All participants (n=15) received written information about the study and signed individual consent forms.

### Short Message Service Text Message Development

Findings from the focus group discussions informed the format (eg, frequency or timing) and content (eg, messaging) for the SMS text messaging intervention. Methods to develop the SMS text messaging strategy also included the following: (1) review of previously published literature, (2) examination of successful SMS text message interventions presented at national and local conferences and events (eg, 2015 Society of Behavioral Medicine Conference, NIH mHealth listserv, and research forums), (3) review of existing spiritually based SMS text messages, (4) review of health-related Biblical scripture, and (5) recommendations from a community advisory board. Frequency and timing of the SMS text message delivery were determined by the preferences and needs of the target audience. SMS text message content was determined before initiation of the pilot study. A total of 18 short project-specific SMS text messages that were health-related and spiritually themed were developed by the PI and initially reviewed by members on the community advisory board. To ensure suitability for delivery via a SMS text message, each message was two to three sentences long.

### Cognitive Response Interviews

Once the spiritually based cervical cancer SMS text messaging educational intervention was developed, cognitive response interviews (n=8) were used to assess the content of the SMS text messaging library, to ensure that the content was acceptable and understandable, particularly for church-attending African American women in the age range of 21 65 years. Cognitive response procedures involve intensive one-on-one interviews in which participants may be asked to think aloud about the content they have read, paraphrase the information, and respond to other planed inquiries and probes [71,72]. On the basis of suggestions from the participants, the research team revised the texting curriculum following findings from the cognitive response interviews. The final product was a programmed spiritually based SMS text message library on cervical cancer prevention and early detection to be delivered over a 16-day period.

### Initial Usability Testing

Initial usability testing of the pilot program then ensued. During the initial usability testing, the PI and the two research assistants (RAs) beta tested the revised system and used this time to identify and correct some initial programming issues. An invitation for beta testing was also extended to members on the community advisory board and to the participants who previously participated in the focus group discussions during phase 1. The spiritually based cervical cancer SMS text messaging educational intervention was then finalized for subsequent feasibility testing (described elsewhere).

### Data Analysis

The focus group discussions and cognitive response interviews were digitally recorded, and the audiotapes were transcribed verbatim. The PI reviewed each of the transcripts for transcription accuracy. Each of the focus group transcriptions was also combined with two sets of observers’ detailed field notes and summary reports.

Data from phases 1 and 2 of the CervixCheck intervention were qualitatively analyzed using standard text analysis. Data-driven content analysis was used to explore the findings with the PI and two trained RAs identifying themes independently. Themes were identified in accordance with the methods described by Owen [73]. In an iterative analytic process, the three researchers independently read and reviewed the transcripts to generate impressions. Together with the research questions that shaped the discussion and interview guides, these impressions then formed the basis of the initial coding framework. Participants from phases 1 and 2 were also extended an opportunity to review the findings and to confirm the main themes and specific phases that demonstrated them.

## Results

### Focus Group Characteristics

The 15 focus group participants ranged in age from 23 to 58 years, with a mean age of 39.57 years (SD 14.17; median 45.50). One participant had less than a high school education, 3 had a high school diploma or general equivalency development credential, 1 had attended some college, 6 had a bachelor’s degree, 3 had a master’s degree or higher, and 1 did not answer the question. Six participants were currently single, 5 were married or living with a partner, 3 were separated or divorced, and 1 was widowed. Nine participants were employed full time, 1 was not employed at the time, 1 was receiving disability, 4 were employed part time, and 1 did not answer the question. One participant reported being a breast cancer survivor, 9 reported having a family history of cancer, and 14 reported having been screened for cervical cancer at some point in their lives, whereas one had not. Of the 15 women who participated in the discussions, approximately half (n=8) indicated that they had undergone a Pap test within the previous 3 years, with 5 out of 8 of these women reporting that they had received their most recent Pap test within the last 12 months. All but one reported having some form of health insurance coverage (Table 1).

### Focus Group Findings and Recommendations

Although general access to health care (eg, having coverage through health insurance or access to a regular doctor nearby) was mentioned as a reason as to why women in their communities may not get screened for cervical cancer, the participants overwhelmingly expressed how the lack of screening within their communities may actually have more to do with the lack of general knowledge, awareness, and communication around this particular type of cancer. Women across both focus groups mentioned how cervical cancer and Pap testing get minimal attention in their community, especially in comparison with other cancers such as breast cancer and prostate cancer. One woman shared how:

...[she] kn[e]w a lot of people who don’t get diagnosed sometimes until it’s too late because a lot of people just don’t think about it. They think of all the other cancers first before they think of cervical cancer.

**Table 1:** Sociodemographic characteristics and Pap test screening behavior of focus group and cognitive response testing participants (N=23).

Sociodemographic characteristics and Pap test screening behavior		n (%)
**Age (years)**		
	21-35	8 (35)
	36-50	10 (43)
	51-65	5 (23)
**Education**		
	Less than high school	1 (4)
	Some high school	0 (0)
	High school graduate or General Equivalency Development	3 (13)
	Some college	5 (22)
	Bachelor’s degree	8 (35)
	Master’s degree or higher	5 (22)
	Missing^a^	1 (4)
**Marital status**		
	Single	9 (39)
	Married or living with partner	7 (30)
	Separated or divorced	6 (26)
	Widowed	1 (4)
**Employment**		
	Full time	11 (48)
	Part time	6 (26)
	Not currently	2 (9)
	I’m disabled	1 (4)
	I’m retired	2 (9)
	Missing^a^	1 (4)
**Insurance**		
	Private	15 (65)
	Public	7 (30)
	None	1 (4)
**Religion**		
	Christian	13 (100)
	Other	0 (0)
**Ever had a Pap test**		
	Yes	22 (96)
	No	1 (4)
**Received a Pap test within the previous 3 years**		
	Yes	14 (64)
	No	8 (36)
**Received their most recent Pap test within the last 12 months**		
	Yes	9 (64)
	No	5 (36)
**Family history of any type of cancer**		
	Yes	15 (65)
	No	4 (17)
	Not sure	4 (17)
**Cancer survivor**		
	Yes	1 (4)
	No	22 (96)

^a^Data are missing for 1 participant because the participant did not answer this question.

Another woman shared:

...you just don’t hear too many people talk about cervical cancer...a lot of people may not think “ok look, let’s go get tested”...how are we supposed to know to go get screened [cervical cancer] if we don’t even hear people around us talking about it?

When asked why the women believed this was the case in their community, one woman stated:

...well, it’s the generations too...the conversations are not there. My mom never talked to me about it. Everything that I’ve learned regarding maintaining my body as a woman...[it was] derived from or what I was exposed to in school and things of that nature...and they didn’t talk about it because she wasn’t comfortable or she didn’t know...I truly believe it is from education, direct communication.

Another woman in the same focus group also shared how:

...well, and there’s the fear of cancer...no way’...I don’t want [the doctors] to tell me that I have it...it’s really important for us to get tested so we know what is going on with our bodies and so that we can know what our options are...

to which the majority of her fellow focus group participants nodded in agreement. Participants in both focus groups agreed that the lack of general knowledge, awareness, and communication about cervical cancer is a contributing factor to why many women in their communities do not often get screened or treated until it is too late.

As the focus group discussions naturally transitioned over to whether or not the participants have ever gotten Pap test screened and what their experiences were like, the following additional themes surfaced: (1) discomfort (eg, most of the participants described getting the Pap test as being invasion, awkward, painful, cold, and uncomfortable); (2) intergenerational relationships (eg, the older generation, the parents, are not discussing these issues with their children; there is a lack of conversations around cervical cancer and Pap testing within the family setting); (3) confusion regarding current screening recommendations (eg, some of the women did not know when you should start getting the Pap test and how often you should get them); and (4) patient or provider relationship and communication (eg, doctors need to actively let their patients know the importance of getting a Pap test, the risk of not getting one, and patiently walk their patients through what the procedure is like). With regards to general knowledge around the Pap test, a little over half of the focus group participants indicated that they were unaware that it was a screening procedure for cervical cancer. One woman emphasized this concern by sharing the following:

...when you came into womanhood, I mean, it was my understanding that part of your normal yearly thing was, you go get a Pap smear, but I never connected it to cervical cancer...I just assumed it was part of my physical exam...now I’m wondering how often I actually got these Pap smears done.

Moreover, there was a lot of confusion across both of the focus groups when the women were asked whether they knew what the screening recommendations currently were for cervical cancer. Although all of the participants from phases 1 and 2 reported familiarity with the Pap test, 83% (19/23) of the women were unable to correctly identify what the current Pap test screening recommendation was for cervical cancer. These individuals were unable to identify when (ie, at what age) a woman should begin to get Pap test screened and how often these screenings should take place.

During the last portion of the focus group discussions, participants were asked to share whether they thought a spiritually based SMS text messaging educational intervention would be appealing for the promotion of cervical cancer early detection among church-attending African American women, and if so, what factors should be considered when developing this type of program (ie, what would the program look like). Across both focus groups, the women enthusiastically shared their support for such a program. For example, one woman responded:

Yes, of course it will because it is something that will provide us encouraging [and] positive messages... just like the ones we get in church. Then, when we get [the messages], we can even share it with the next 10 ladies on our list...pass it right around.

Another woman from the same focus group added:

I know for our church, we have been doing a lot with making health a priority. Just having [that] information has helped our church tremendously...so, I would agree with her that having similar messages and reminders like that, even when we are away [from the church setting], is just a plus.

Women who expressed positive attitudes regarding the intervention’s potential ease and convenience were often drawing comparisons to their prior experiences with similar SMS text messages that they were already getting from their congregation and health care providers (HCPs). Statements comparable with the ones shown above were always followed along with the majority of our participants nodding in agreement and elaborating on the topic.

Additionally, the women in our focus groups also expressed how a spiritually based SMS text messaging intervention to promote any health topic would “definitely get [their] attention” because “it is just a natural fit.” When we probed further and asked our participants what they meant when they refer to an intervention, such as ours, being a “natural fit,” one participant shared:

Well, you know right...God, the Bible, our religion...all of that is important for so many of us. That’s why we are in church...we go to church. Getting religious prayers [and] reminders, if you can tie that back to something else important to us, like our health, you get our attention. You know? Like, I mean, why not?

Another woman, who was slightly older, also responded positively about why such a program would be a “natural fit,” stating the following:

Yes! It absolutely is [a natural fit] because it will just add to what my church is already doing...my church, you know, we have helping and healing...and they have brought up cervical cancer prevention before.

The general consensus was that an intervention that is actually spiritual in nature, through message framing or content, would be more effective for women like themselves. One young woman stated:

Even if I didn’t care right away [about cervical cancer prevention], when the text pops up on my phone, you know...it’ll just catch my attention right there and then...I already have something like that called InstaPray. It has prayers and spiritual messages so, of course, if it was something like that, I would definitely use it. It’s like a mix of Instagram and Twitter. You can change how many notifications you get too!

Other participants in that focus group all nodded in agreement, acknowledging that when spiritual individuals such as themselves are presented with spiritual messages, their receptivity and perceived relevance of the underlying health topic is increased. They overwhelming voiced support for a spiritually based SMS text messages on cervical cancer prevention because of their comfort and familiarity with religion already being a large part of their everyday lives. They perceived benefits associated with not only early detection and the need to get checked, but also with the concept of how faith can be used to get them through their life experiences and to ease any anxiety that a woman may feel when “the big C” is mentioned, if ever directly. Considering and getting a Pap test was seen as “easier” when one is calmed by their faith. This prompted a number of our focus group participants to tell personal stories of either their own or a loved one’s illness and how their religion and faith helped them through the varied experiences.

Understanding the role of faith in cancer screening and whether these women desired a spiritually based intervention through these focus groups, reaffirmed what we had learned from our previous studies [32,33,39]. However, to further grasp what influences our participants to go in to get their Pap test and the type of channels, message framing, and content that would specifically work for them, we continued to probe our participants and asked them to elaborate on how they would go about educating and encouraging individuals, such as themselves, to get Pap test screened. They were initially asked about their current access, use, and preference for various technology-based programs and platforms (Table 2).

All participants in the focus groups had text-capable phones, with 87% (13/15) indicating that they had unlimited usage plans. The participants overwhelmingly reported cell phone use and an interest in receiving spiritually based SMS text messages on cervical cancer prevention.

The participants were also asked to share their thoughts on what they felt would be the best way to deliver information about cervical cancer and the benefits of getting Pap smear screened as recommended and whether incorporating technology to some extent was a reasonable idea. Without hesitation, the majority (87%, 13/15) of the participants across the two focus groups ecstatically agreed that utilizing SMS text messages would be a great way to quickly get short health-related educational messages across to individuals in their community. Two women shared the following views:

...everyone has a [cell] phone and texts these days...like my mom texts me a lot...telling me to come over dinner or to remember to do something...she doesn’t text as much as my daughter, but she does text...and come on, she’s 62!…She still doesn’t know how to use our tablet or computer though...

...who doesn’t text? I may not [send] text much...you know, I’m almost 53, but I get them often. I get [text messages] from my family, church groups...and I like the ones that they send me about the traffic...or storms...and now my doctor, my dentist...I like those! I also like how if I forget or need to find something, I can go back to check some of my old texts to see if the information is still there.

Further recommendations from the focus group discussions regarding what an SMS text messaging–based program would look like included (1) Having a catchy project name that reflects the topic at hand, (2) Making sure the SMS text messages were not going to get delivered too early in the morning (ie, before 8 AM) or too late in the evening (past 8 PM), (3) Incorporating testimonials from women such as themselves or from famous celebrities that they can identify with, (4) Balancing the health-related educational messages with other messages that were broadly more positive and motivational in nature, (5) Ensuring that the faith-based messages included direct scriptures that most church-attending individuals can quickly recognize, and (6) Including local resources where individuals can go get screened for cervical cancer, especially if health insurance coverage is not a possibility.

**Table 2:** Technology access, use, and preferences for participants in the focus group discussions and cognitive response interviews (N=23).

Technology access, use, and preferences		n (%)
**Contact preference**		
	Cell phone	22 (96)
	Home phone	1 (4)
**Do you use a cell phone?**		
	Yes	23 (100)
**Is your phone a “smartphone”?**		
	Yes	17 (74)
	No	6 (26)
**Do you use a cell phone for text messaging?**		
	Yes	23 (100)
**How often do you use text messaging to communicate?**		
	More than once a day	18 (78)
	Once a day	2 (9)
	2-3 times a week	2 (9)
	Once a week	0 (0)
	Less than once a week	1 (4)
**Do you have a computer at home with internet?**		
	Yes	19 (83)
	No	4 (17)
**Do you have a Facebook account?**		
	Yes	15 (65)
	No	8 (35)
**Do you have a Twitter account?**		
	Yes	10 (44)
	No	13 (57)

Although participants indicated the popularity of unlimited SMS text messaging plans within their communities, they stressed that the research team should never send out more than two messages per day. There were mixed feelings regarding receiving daily messages from the project. Some individuals indicated that they would appreciate the regularity and consistency of getting the daily messages, whereas others indicated that “it would be too much for me” or that “I kind of would like a break here and there.” In general, participants indicated that sending out several messages, a few days each week, would be completely acceptable, as long as it did not exceed two per day and that at least one of these two messages (within the same day) was not health-content heavy. To have some balance and to keep individuals such as themselves engaged, participants stressed the importance of having at least one of the messages be a more general spiritually or motivationally based message.

A final recommendation that surfaced during the discussions revolved around the project name. Though reasonably appealing, the originally proposed project name was ultimately replaced based on a suggestion that came from one of the focus group participants. A proposed project name, CervixCheck, was favored by the majority of the participants across both focus groups, as well as all of the participants during the cognitive response interviews. All of the women in the first focus group enthusiastically nodded in agreement when one of their fellow participants stated:

...you need a sexy, like a catchy [project] name, something that gets straight to the point and tells us...we need to get specifically checked for cervical cancer...it’s just something we don’t really ever hear or talk about...but I mean, it’s really important.

As opposed to having a generic project name stressing the idea of overall women’s health, the appeal with the new project name was that it actually reflected the specific type of cancer that the project was trying to target.

### Initial Draft of the Text Messaging Library

Through the focus group discussions, messages and wording preferences and recommendations for incorporation into the SMS text messages were recorded. On the basis of the information collected, the research team developed a 14-day one-way SMS text messaging pilot intervention. The recommendations of the focus groups and the advisory board were reviewed by the investigative team and used, along with a review of existing cervical cancer educational materials, to develop draft content for the SMS text messages to be used in the intervention. The originally drafted SMS text messaging library comprised a total of 18 messages. The 18 draft messages included the welcome and closing messages to participants, as well as nine health-specific messages and seven spiritually based messages. Core content covered areas such as the definition of cervical cancer, cervical cancer’s impact on the African American women population, the role of Pap testing in cervical cancer prevention and early detection, and information on where individuals can go for free or low-cost screening in their local communities. The spiritually based messages involved themes such as being a good steward over the body as a gift from God; personal responsibility for the life and body, which is a gift from God; being healthy so that one can serve God and those important around her; use of faith to get through cervical cancer screening; God will take care of us, but we must do our part and get screened; and various scriptures supportive of health. Selective examples of messages from the initial draft of the SMS text messaging library are shown in Table 3.

### Cognitive Response Testing Participant Characteristics

The 8 cognitive response testing participants were in the age range of 21 to 65 years, with a mean age of 41.67 years (SD 18.77; median 41.67). Four of the participants had attended some college, 2 had a bachelor’s degree, and 2 had a master’s degree or higher. Three of the participants were currently single, 2 were married or living with a partner, and 2 were separated or divorced. Two of the participants reported that they were retired, 2 were employed full time, 1 was not employed at the time, 2 were employed part time, and 1 individual did not answer the question. None reported being a cancer survivor, and only 6 reported having a family history of cancer. All participants reported having been screened for cervical cancer at some point in their lives, and everyone also indicated that they had some sort of health insurance coverage during the time of their individual cognitive response interviews. Of the 8 women who participated in the cognitive response interviews, 75% (6/8) indicated that they had undergone a Pap test within the previous 3 years, with 4 out of 6 of these women reporting that they had received their most recent Pap test within the last 12 months.

### Cognitive Response Testing Recommendations

Participants understood and found acceptable the vast majority of the content that was tested. A consistent concern that surfaced during the interviews was one about the first educational message (health 1) and when it should actually be presented in the program. Participants did not like the idea that the very first educational message from the project would be one that hones in on the devastating impact that cervical cancer has on women in their community. Although all of the women acknowledged the importance of including such a message, they did not feel that it was appropriate to start with a message that “invokes fear” or “is depressing.”

**Table 3:** Selected examples of messages from the draft short message service (SMS) text messaging library.

Text order/#	Message type	Construct	Key message	Message text	Character # (without spaces)
1	Start	Welcome	Thank you for enrolling	(CervixCheck) Hi, (first name of participant). Thank you for being part of the “CervixCheck” Women’s Health Project. If you are still interested in receiving text messages over the next two weeks from the “CervixCheck” project about cervical cancer, please reply to xxxxx with the response “YES.”	276
2	Health 1	Knowledge	Impact; rates or statistics	African American women are at higher risk of dying from cervical cancer than other women. This is because too often the cancer is found later, after it has spread.	163
3	Spiritual 1	Knowledge	Taking care of health	“My People are destroyed from lack of knowledge.”—Hosea 4:6	60
9	Spiritual 4	Subjective norms	Responsibilities	When it comes to our health, doing “our part” means that we take care of our bodies in general, and get the routine exams that we need. This includes getting a Pap test—the part we do so that God can do His part.	215
13	Spiritual 6	Perceived behavioral control	Self-motivation to take action	“I can do all things through Christ which strengtheneth me.”—Philippians 4:14	78
15	Health 8	Cues to action	Resources: link to more information or free services	When are you due for your routine Pap test? Talk to your doctor to find out. No insurance? No problem. For more information and to see if you are eligible for free screening, go here: (short url).	195
16	Health 9	Social network	Spread the word; intergenerational communication	Spread the word and pass the wisdom down from one generation to the next. Share this information with the next generation like a good family recipe.	148

**Table 4:** Scheduling of short message service (SMS) text messages for the CervixCheck pilot intervention. N/A: not applicable.

Message #	Day	Day of week	Time	Interactive activity
1	1	Saturday	12:00 PM	Request for response 2 opt-in
2	1	Saturday	8:30 PM	N/A
3	2	Sunday	2:00 PM	N/A
4	2	Sunday	5:00 PM	N/A
5	4	Tuesday	12:00 PM	Link to supplemental website
6	4	Tuesday	4:00 PM	Link to image
7	5	Wednesday	12:00 PM	“True or false” question posed (prompt for a close-ended response); website link
8	6	Thursday	12:00 PM	N/A
9	6	Thursday	4:00 PM	Link to supplemental website
10	8	Saturday	12:00 PM	N/A
11	8	Saturday	4:00 PM	N/A
12	9	Sunday	2:00 PM	“Thoughts?” (prompt for an open-ended response)
13	9	Sunday	5:00 PM	“Agree or disagree” question posed; (prompt for an open-ended response)
14	11	Tuesday	12:00 PM	N/A
15	11	Tuesday	4:00 PM	Link to testimonial
16	12	Wednesday	12:00 PM	Link to testimonial
17	13	Thursday	12:00 PM	N/A
18	13	Thursday	4:00 PM	N/A
19	15	Saturday	12:00 PM	Link to resources
20	15	Saturday	4:00 PM	N/A
21	16	Sunday	2:00 PM	N/A
22	16	Sunday	5:00 PM	(Prompt for further questions)

One woman shared:

...[how] you should move this [message] to later in the program...you want to start with a more uplifting message...you want to catch our attention about cervical cancer and how it affects people like me, but an initial message like this would totally turn me off...it’s important but it just sounds too scary.

Another concern that arose during phase 2 was how there was still some confusion as to where the cervix was and what the Pap test procedure included. To remedy these concerns, participants recommended that we include direct links to images and/or videos that would elaborate on the anatomy of the women’s reproductive area. One woman explained:

...for those of us who want more information, at least you can have it right there and easily accessible...even if we don’t look at what you send us right away, at least it’ll be in our phones and we can return to it when we feel like it.

Other recommendations included the need to “really bring in the personal stories” and to place an emphasis on testimonials from women such as themselves. Some of the participants also provided direct edits on how to the research team could condense some of the draft messages that originally ran beyond the 160 characters limit. As system specifications limits text content to 160 characters and spaces, our drafted messages needed to be redesigned to be concise. Finally, a message “sign-on” was recommended for any text that the project team was planning to send out. There was general consensus across the interviews that some sort of project branding was necessary to let participants know which messages were directly coming from the research team. It was also noted that a message “sign-on,” as opposed to a message “sign-off,” would be ideal because “you want [us] to know right away that that incoming message is from you, your project...If you start each message with your project’s name, participants can get in the habit of recognizing them as soon as they come.” Beyond the recommendations mentioned above, other materials tested during phase 2 were found to be both understandable and acceptable, including the spiritual themes and scriptures.

In response to feedback from the participants in phase 2, we revised the texting curriculum to include four additional messages. The 22 “final” messages include the welcome and closing messages to participants, as well as 10 health-specific messages, four spiritually based messages, and six messages that were both health-specific and spiritually based in nature (Table 4). The messages now span across 16-days, averaging at about 11 texts per week, with no more than two messages scheduled for delivery per day.

The welcome or opt-in message is scheduled to take place on the Saturday before the first program message is sent off the next day (ie, on a Sunday). During the weekdays and on Saturdays, messages are scheduled for delivery at 12 PM (around lunch time) and/or 4 PM (before the end of a regular work day’s shift). On Sundays, messages are scheduled for delivery at 2 PM (after most church services) and 5 PM (before dinner time). “Off days” are scheduled for every Monday and Friday, days of the week in which participants indicated that they would be “more swamped” and that it would not be ideal to receive program-related information. The closing message is also scheduled for delivery on a Sunday.

## Discussion

### Principal Findings

This paper reports on the design and development of the CervixCheck project, a spiritually based SMS educational pilot intervention aimed at increasing cervical cancer awareness and Pap test screening intention among church-attending African American women aged 21 to 65 years. This intervention situates health beliefs and behaviors in the context of culture and information technology [33,74]. Previous research has suggested that the development and implementation of culturally appropriate interventions through a community-based or community-engaged approach can be successful in addressing the underutilization of cancer screening among African Americans [33,75-77]. The process in developing this intervention involved substantial participation of the priority population in all stages of the intervention development. This participation is viewed as a necessary element of a culturally appropriate intervention, not only to allow for community ownership of the project but to ensure that the intervention is indeed culturally appropriate, and not based on assumptions from the research team that may or may not be accurate.

The findings showed that a culturally appropriate SMS text messaging intervention should be developed based on the target population’s perspectives and input. The intervention development required collecting data from the participants regarding both the content and delivery formats of the culturally relevant health messages. In general, the participants felt that a cervical cancer educational program, framed within a spiritual context, was a good and innovative idea. The spiritual concepts generated by this group of participants were quite similar to those generated in previous cancer screening educational interventions, especially in qualitative projects examining spirituality and health beliefs [33,78,79]. To be additionally effective with the target population, the SMS text message content also needed to be encouraging, empowering, and thought-provoking, all while being short, informative, and direct. The focus group discussions suggest that the messages should focus on raising awareness and increasing general knowledge and acceptance of the Pap test to change attitudes, possibly before any specific behavior change [80,81]. There was also a general preference for the inclusion of culturally appropriate visual and motivational messages that emphasizes one’s role in relation to God, family, community, and women such as themselves. Overall, these findings are consistent with the Centers for Disease Control and Prevention’s suggestions that to quickly engage the reader, messages need to be clear, give important information first, be action-based, and easy to understand [81,82].

Although expansive reviews of the literature describe the weight of the evidence of the relationship between spirituality and health as being largely positive [27,83], not all religious influences are positive, or adaptive, in nature [84-87]. For example, one negative aspect of religious involvement is the idea that some individuals believe that illness may be the result of punishment for their wrongdoings or sins [88-90]. Individuals who defy religious norms may experience feelings of shame or guilt, or they may fear punishment from God [89]. Research on this particular notion has suggested the idea that serious illnesses such as cancer may be viewed as being the consequence of punishment for sin [30,91,92] and thus, may translate to engagement in maladaptive health behaviors such as forgoing cancer screenings [89,90]. With regard to implications from this study that addresses such situations, where religion might act as a barrier for a woman to undergo a gynecological examination, this is where the development, implementation, and evaluation of a two-way spiritually based SMS text messaging intervention that incorporates counseling may play a role in working with individuals who may hold such beliefs, with a spiritual sensitivity and competence [93].

### Conclusions

As mobile technology become more popular and advanced, a culturally appropriate SMS text messaging intervention could be an effective medium to deliver sensitive health information and eventually promote positive health behavior in underserved population. Studies investigating SMS-based interventions in minority populations have recommended more extensive research to better understand the most effective content of text messages to increase the benefits derived from mHealth apps [94-96]. This paper reports on formative research conducted to inform the development of an automated one-way SMS text messaging intervention to disseminate cervical cancer prevention and early detection education. The development of the SMS text messages not only involved consideration related to the content of the messages but also with technological specifications. The findings from phases 1 and 2 of this study show the importance of obtaining feedback about the content of SMS text messages and of pretesting the SMS text messaging distribution system before further implementation should take place.

SMS text message interventions should be carefully developed, tested, and refined before implementation, to ensure they are written in the most appropriate way for their target population. Although some may be tempted to rely on common sense and skip a formative stage before implementation of interventions, the process of iterative formative research to develop the content and logistics for developing this program was indispensable to identify challenges to be addressed before the implementation of the piloting phase (phase 3). This research provides insights into the appropriate number of messages to consider, the timing of when they should ideally be sent, and the educational content for consideration in an SMS text message–based intervention to promote cervical cancer prevention and early detection information for African American women. Message development research is important for effective interventions, and public health practitioners need to pay close attention to how the messages will be received by the recipients.

The development, and ultimately the implementation and evaluation, of this CervixCheck spiritually based SMS intervention will provide important findings into what may be considered an overlooked minority population and missed opportunity in health information technology research [45,56-60]. Although there is a growing body of literature reporting positive outcomes of SMS-based communication with STIs and cancer prevention, there is still very little research about the integration of communication technologies with previously reported effective intervention approaches such as being spiritually based. By using important spiritual themes to frame cervical cancer educational content and by delivering these health messages through a popular communication delivery channel for this targeted group, cancer interventions can move one step closer to being more accessible and culturally appropriate for the African American women community.

### Limitations

There are, however, some limitations to the approach used during the formative phases of this study. First, the process of working with church-attending African American women on message design and refinement was fluid and often nonlinear. The PI gave up her own sense of control over the project as it evolved into a partnership endeavor. Second, although the PI conducted quality control measures, the findings from this study may still be at risk for social acceptability bias. The focus group discussions and cognitive response interviews were conducted on the culturally sensitive topic of cervical cancer and Pap testing. This may have deterred our participants from sharing their true thoughts and feelings.

Third, despite efforts to recruit individuals with varying sociodemographic characteristics, participants from the focus group discussions and cognitive response interviews had fairly high education, with 74% having at least an undergraduate degree (17/23). Furthermore, the majority (96%, 22/23) of the participants indicated that they had some sort of health insurance coverage during the time that this study was conducted and that they had already been screened for cervical cancer at some point in their lives. The final make up of our convenience sample showed us that we were barely able to reach some specific subgroups, in particular unscreened African American women whose experiences would have enriched our findings. Thus, the lack of representativeness from the larger population of church-attending African American women may have potentially limited the generalizability of the findings. This could also, however, reflect the current nature of cervical cancer screening practices among church-attending African American women from Prince George’s County, MD.

Finally, although the authors acknowledge the important role that the HPV vaccine holds in cervical cancer prevention, this study did not heavily focus on this approach. Even women who were vaccinated when they were younger need regular Pap test screening because the vaccines do not protect against all cervical cancers. Additionally, previous studies have suggested that African American women are less accepting of the HPV vaccine [97-99]. Future studies should evaluate the acceptability of the HPV vaccine among the African American community and explore the feasibility of promoting HPV vaccine educational content through mobile-based technology.

Albeit the limitations presented here, it is important to note that the nonrandom sampling design for this study was also purposefully selected in consideration of cultural issues. As African Americans put high values on social relationships, being invited to participate in a study by people with whom they are familiar (eg, health ministry leaders, HCPs who work in the African American community, community health advisors, and/or organizational partners from the already existing community network) was proposed as a more of a reasonable recruitment approach than being contacted by a third-party telephone interviewer they do not know. Although this study utilizes a convenience sample, this culturally sensitive sampling strategy serves as the initial step to create some degree of capacity building among the African American women community and will ideally create a sustainable infrastructure to support future research and cervical cancer intervention programs similar to this one.

In the next phase of this project, we will pilot-test the CervixCheck program and will use baseline and follow-up surveys to assess the program feasibility, acceptability, and initial efficacy. The findings from this intervention will inform future research and practice in developing culturally appropriate health communication approaches for church-attending African American women. If this pilot intervention shows feasibility, acceptability, and initial efficacy for increasing cervical cancer awareness and Pap test screening intention, such a program can be adapted or further expanded and evaluated for its effectiveness in its contribution to the elimination of cancer disparities that negatively impact African American communities. Future studies using a more rigorous research design with a larger sample of African American women (eg, a randomized controlled trial with multiple follow-up time points where actual screening behavior can be taken into consideration) is therefore needed to validate the effectiveness of interventions such as the CervixCheck program.

From a public health standpoint, this study also informs the work of researchers engaged in efforts to meet the *Healthy People 2020* objectives to reduce the death rate from cancer of the uterine cervix (C-4) and to increase the proportion of women who are counseled by their providers about Pap tests (C-18.2). *Healthy People 2020* is a 10-year health agenda released by the US Department of Health and Human Services that is designed to guide national promotion and disease prevention efforts to improve the health of all people in the United States [100]. Technology-based platforms can, therefore, provide researchers the ability to reach a large number of people at a relatively low cost, which can ultimately lead to a greater public health impact with regard to cervical cancer early detection health promotion efforts.

## Abbreviations

HCP: health care provider
HPV: human papillomavirus
mHealth: mobile health
NIH: National Institutes of Health
PA: physical activity
Pap: Papanicolaou
PI: principal investigator
RA: research assistant
SMS: short message service
STI: sexually transmitted infection
